# *QuickStats:* Changes* in Late Preterm Birth Rates,^†^ by State — National Vital Statistics System, United States, 2014 and 2016

**DOI:** 10.15585/mmwr.mm6724a7

**Published:** 2018-06-22

**Figure:**
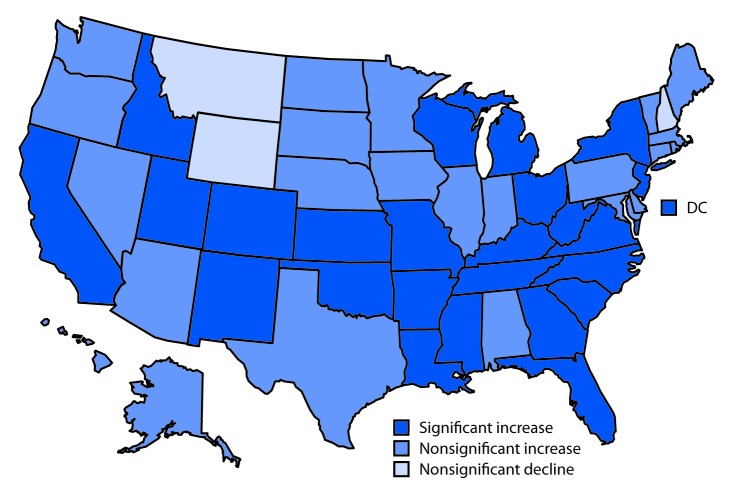
The late preterm birth (34 to 36 weeks of gestation) rate rose 4.0% in the United States, from 6.82% in 2014 to 7.09% in 2016. Increases in late preterm birth rates occurred in 24 states and the District of Columbia during 2014–2016. Increases in an additional 23 states were not statistically significant. Nonsignificant declines in late preterm births were observed in three states.

**Source:** National Vital Statistics System, Natality, 2014 and 2016. https://www.cdc.gov/nchs/data/nvsr/nvsr64/nvsr64_12.pdf and https://www.cdc.gov/nchs/data/nvsr/nvsr67/nvsr67_01.pdf.

